# Joy Shumake-Guillemot: linking climate to health

**DOI:** 10.2471/BLT.23.030223

**Published:** 2023-02-01

## Abstract

Joy Shumake-Guillemot talks to Gary Humphreys about the benefits of intersectoral, climate and health initiatives and the potential of climate services for the health sector.

*Q: How did you come to focus on the connections between climate and health?*

A: I was raised in rural Missouri (United States of America) and grew up very aware of the influence of seasons and the environment on my local community, including the impact on people’s health. So that link was made before I entered academia or started my humanitarian work. That said, there was a “light bulb” moment for me. It happened in 2002 when I was working in Somalia during one of the country’s devastating droughts. We were dealing with a cholera outbreak, and one afternoon, at a debriefing, a community elder spoke up and said, “Well, you know, when the river gets so low, this just happens.” It was a very simple statement about a predictable event that combined considerations of climate, the ecosystem and health – and it got me thinking about the possibility of developing early warning systems based on meteorological and ecosystem phenomena. I started asking myself how – in view of likely climate change acceleration – we can make use of the science coming out of the fields of climate, meteorology and environment in a more systematic way in public health. That was the seed of work that later went into the development of the joint programme between the World Health Organization (WHO) and the World Meteorological Organization (WMO), as well as the *Operational framework for building climate resilient health systems* that was launched by WHO in 2014.

*Q: What do you mean by a “climate resilient health system”?*

A: A health system that has the capacity to anticipate problems and continue to function when they arrive – not becoming disrupted by extreme weather events, for example. Climate resilient health systems have the capacity to recognize, monitor and disseminate information regarding climate-related risks, as well as the capacity to mitigate and respond to them. Another defining aspect of resilience is the capacity to learn from experience and to use that learning to drive iterative improvement.

*Q: You mention the development of an operational framework. Can you describe it in simple terms?*

A: The framework offers ministries of health a structured approach to understanding how climate can impact different aspects of the health system and how to address challenges while making the most of opportunities for developing resilience. It is based on the health system building blocks construct (an analytical framework developed by WHO that describes health systems in terms of six core components) and is therefore familiar to policy-makers and decision-makers. Since 2014, the framework has become central to WHO climate adaptation work and is what countries are encouraged to use as an organizing approach to their national health adaptation plans. Today, around 80 countries have implemented health adaptation plans which reflect the operational framework to different degrees, but it must be said that the health sector in general has been slow to respond to the risks identified by the climate science community and there is an urgent need for more dialogue and collaboration.

*Q: Can you say more about that?*

A: For example, there is still a tendency to work in silos, with actors in the health space going it alone, rather than working across sectors. There is also a bias towards investing in reactive response systems, rather than in prevention. Fortunately, this is starting to change, the rise in prominence of the One Health framework being a good example of cross-sectoral thinking with a focus on preparedness and prevention. There’s also increasing interest on the part of the health community in climate science and technology such as remote sensing satellite imagery and mobile technologies, not to mention potential applications of artificial intelligence.

*Q: To what extent can remote sensing information replace observations made on the ground?*

A: Remote sensing information remains a proxy to what is really occurring locally and may not always be sufficiently robust to make local decisions, but it is a valuable complement and can provide large-scale/transboundary perspectives. 

*Q: How comprehensive is on-the-ground data capture in lower-income countries?*

A: As with health surveillance data, it’s never perfect, but in fact, globally, even in low- and middle-income countries, sophisticated infrastructure for ground level weather observations exists.

*Q: What can be done to promote more health and climate collaboration?*

A: To begin with, we need a new generation of scientists and professionals who are trained to speak and understand the languages of both climate and health. At the national and transnational level we also need stronger collaboration between institutions, supported by the creation of specific joint positions, projects and coordination mechanisms. WHO and WMO have shown the way in this regard by launching an interagency collaboration on the implementation of the Global Framework for Climate Services in 2009 and a Joint Climate and Health Office in Geneva, Switzerland in 2014. A new 10-year plan (2023–2033) is just being finalized and will hopefully further accelerate policy integration and capacity development, while also supporting the development of new technical networks and partnerships. Anticipated outputs include climate services and products, research and communications.

*Q: What do you mean by climate services and products?*

A: Climate services underpin the delivery and support of climate products, which include tailored climate outlooks, impact-based climate forecasts, vector suitability maps (maps designed to elucidate relations between known disease vectors such as mosquitoes and climate change), health early warning systems, risk assessments, etc. These services and products derive from cross-disciplinary partnerships aimed at using knowledge about the climate and weather to inform decisions in climate-affected sectors, such as agriculture or health. Health professionals interested in applying climate science often struggle to access, interpret and use climate and weather data, in the same way an expert from another field might be challenged to appropriately interpret and use epidemiological data. By bringing together climate and health expertise, climate services can help with this.

*Q: Is the concept of climate services widely understood in the health sector?*

A: When I set up the joint programme, one of the first things I did was to put out an open call to stakeholders in the global meteorology and public health communities to ask them about their views and experience with climate services for health. The response I got included around 200 case studies which revealed considerable interest but also a number of important misconceptions. It was in response to that exercise that we published *Climate services for health: improving public health decision-making in a new climate *which was released as a joint publication of WHO/WMO in 2016. It contains a common framework, definitions, and 40 case studies showing the range of services available and how they can be applied.

“We need […] scientists and professionals who are trained to speak and understand the languages of both climate and health.”

To further help health partners understand and make use of climate science and services, we also recently launched a new global open-access web-based platform – ClimaHealth.info – to serve as a go-to technical reference point on climate and health for a wide range of users – from policy-makers to community groups. Site users can tap into curated guidance and research documents but can also connect with global experts, and more easily find relevant tools, resources and opportunities. The platform will be enhanced with new content and dynamic features in the coming years and we’re hoping that it will serve to drive learning and cross-pollination between the two fields.

*Q: Can you point to areas where climate and health technical collaboration is already happening?*

A: Collaboration around extreme heat is one. Worldwide, public health agencies are realizing the severity of heatwaves is rapidly increasing and they are actively reaching out to meteorological agencies for support. Over the last five years we have set up a robust global community of practice, supported by WHO, WMO and the US government to help develop these partnerships. Having the WHO and WMO nameplates at the table has really helped bring in public health agencies, ministries of health and meteorological services along with academic institutions, and nongovernmental organizations from many sectors – all of whom want to talk about common goals, targets and concerns. They are also sharing experiences about research they are conducting, how they're deploying heat health early warning systems, and implementing heat action plans. 

*Q: Do you feel that we are prepared for the climate-change-related health challenges to come?*

A: No, but I believe that leveraging climate science in the design and implementation of climate resilient health care policy and effective climate services can go some way to mitigating the shocks that we are probably going to face. We in the interagency office are certainly committed to making that a reality. This year WMO is preparing a report on the state of climate services for health which will, I believe, raise awareness regarding the opportunities available, as well as expose the scale of investment needed for the health sector to truly take advantage of climate science advancements which – and this is something I’d like to stress – really are considerable. Notwithstanding the challenges faced on the ground in some low- and middle-income countries, we have reached a point where we can predict climate and weather events with increasing accuracy and with longer lead times. There is thus a significant opportunity for the health community to harness that capacity and the health-relevant intelligence it can generate. I’m pleased to say that there are clear indications of growing interest in the topic, including the interest shown by health leaders in the new Alliance for Transformative Action on Climate and Health which WHO launched at COP26. The Alliance is bringing together around 75 countries to boost investment in climate resilient health systems and shine a light on the role the health sector can play in climate change mitigation, notably by reducing its own carbon footprint. It is my hope that stronger climate action by the health sector will also trigger greater investment in climate action.

